# Evidence for the utility of cfDNA plasma concentrations to predict disease severity in COVID-19: a retrospective pilot study

**DOI:** 10.7717/peerj.16072

**Published:** 2023-09-18

**Authors:** Katharina Hoeter, Elmo Neuberger, Susanne Fischer, Manuel Herbst, Ema Juškevičiūtė, Kira Enders, Heidi Rossmann, Martin F. Sprinzl, Perikles Simon, Marc Bodenstein, Michael Schaefer

**Affiliations:** 1 Department of Anaesthesiology, University Medical Centre of the Johannes Gutenberg-University, Mainz, Germany; 2 Department of Sports Medicine, Disease Prevention and Rehabilitation, Johannes-Gutenberg Universität Mainz, Mainz, Germany; 3 Institute of Medical Biostatistics, Epidemiology and Informatics, University Medical Centre of the Johannes Gutenberg-University, Mainz, Germany; 4 Institute of Sport Science and Innovations, Lithuanian Sports University, Kaunas, Lithuania; 5 Institute of Clinical Chemistry and Laboratory Medicine, University Medical Centre of the Johannes Gutenberg-University, Mainz, Germany; 6 Department of Internal Medicine I, University Medical Centre of the Johannes Gutenberg-University, Mainz, Germany; 7 Focus Program Translational Neurosciences (FTN), Johannes Gutenberg-University, Mainz, Germany; 8 Research Center for Immunotherapy, University Medical Centre of the Johannes Gutenberg-University, Mainz, Germany

**Keywords:** COVID-19, Sars-CoV-2, Cell free DNA, Organ dysfunction, Clinical outcome

## Abstract

**Background:**

COVID-19 is a worldwide pandemic caused by the highly infective SARS-CoV-2. There is a need for biomarkers not only for overall prognosis but also for predicting the response to treatments and thus for improvements in the clinical management of patients with COVID-19. Circulating cell-free DNA (cfDNA) has emerged as a promising biomarker in the assessment of various pathological conditions. The aim of this retrospective and observational pilot study was to investigate the range of cfDNA plasma concentrations in hospitalized COVID-19 patients during the first wave of SARS-CoV-2 infection, to relate them to established inflammatory parameters as a correlative biomarker for disease severity, and to compare them with plasma levels in a healthy control group.

**Methods:**

Lithium-Heparin plasma samples were obtained from COVID-19 patients (*n* = 21) during hospitalization in the University Medical Centre of Mainz, Germany between March and June 2020, and the cfDNA concentrations were determined by quantitative PCR yielding amplicons of long interspersed nuclear elements (LINE-1). The cfDNA levels were compared with those of an uninfected control group (*n* = 19).

**Results:**

Plasma cfDNA levels in COVID-19 patients ranged from 247.5 to 6,346.25 ng/ml and the mean concentration was 1,831 ± 1,388 ng/ml (± standard deviation), which was significantly different from the levels of the uninfected control group (*p* < 0.001). Regarding clinical complications, the highest correlation was found between cfDNA levels and the myositis (*p* = 0.049). In addition, cfDNA levels correlated with the “WHO clinical progression scale”. D-Dimer and C-reactive protein (CRP) were the clinical laboratory parameters with the highest correlations with cfDNA levels.

**Conclusion:**

The results of this observational pilot study show a wide range in cfDNA plasma concentrations in patients with COVID-19 during the first wave of infection and confirm that cfDNA plasma concentrations serve as a predictive biomarker of disease severity in COVID-19.

## Introduction

Coronavirus disease 2019 (COVID-19) is the greatest worldwide pandemic of the 21^st^ century caused by the highly infectious SARS-CoV-2. According to the World Health Organization (WHO), SARS-CoV-2 has caused more than 768.2 million cases worldwide to date (WHO, 2022b). Although COVID-19 vaccines are being developed rapidly, compared to traditional vaccines, and have been approved worldwide (Kashte et al., 2021), the ongoing COVID-19 outbreak is placing an enormous strain on healthcare resources and poses an extraordinary threat to global public health (Hu et al., 2020). In addition, new SARS-CoV-2 variants with increased transmission rates have emerged in the recent years, further complicating the situation (van Oosterhout et al., 2021). Disease caused by SARS-CoV-2 infection ranges from asymptomatic to mild course of illness to extensive inflammation with severe respiratory disease, multiple organ failure and death. Pulmonary manifestations are common, ranging from cough to pneumonia and acute lung failure (ARDS). Hematological and immune system-related changes such as thrombocytopenia and dysregulation of blood coagulation have been reported (Al-Samkari et al., 2020). In addition, neurological manifestations (Wang et al., 2020), acute kidney failure (Yang & Yang, 2020) and gastrointestinal symptoms such as nausea and vomiting, diarrhea, and gastrointestinal bleeding are associated with COVID-19 disease (Cheung et al., 2020). The WHO considers age $\ge$60 years or health conditions such as lung or heart disease, diabetes or conditions affecting the immune system to be risk factors for severe course of disease (WHO, 2022a). There are strong recommendations for corticosteroid treatment in moderate cases, and recommendations for immunomodulatory treatment with Baricitinib and Tocilizuman in patients with pneumonia. However, to date there is no universally proven effective antiviral therapy for COVID-19 patients and no reliable laboratory parameters to monitor therapy. Therefore, the only life-saving therapy in severe cases is bridging-to-recovery, *i.e*., organ support or replacement in the event of organ failure.

Three years into the pandemic and despite the availability of effective vaccination the incidence of SARS-CoV-2-infection continues to show a wavy trend with potentially serious consequences in almost all age groups. Therefore, reliable biomarkers are still required. Biomarkers are not only needed for prognosis, but also for predicting response to treatment and thus for improving in the clinical management of patients with COVID-19. Recent studies have linked laboratory measures of hyperinflammation such as macrophage chemoattractant protein 1 (MCP-1), C-reactive protein (CRP) and interleukin-6 (IL-6), ferritin and procalcitonin (PCT) as strong predictors of disease severity in hospitalized patients with COVID-19 (Broman et al., 2020; Jøntvedt Jørgensen et al., 2020; Mishra et al., 2022). Another promising, non-invasive biomarker of COVID-19 severity from liquid biopsy is cell-free DNA (cfDNA), which is passively released after cell damage and/or actively released from hematopoietic (immune) cells (Dwivedi et al., 2012; Huttunen et al., 2011). Increased levels of cfDNA have been detected in various pathological conditions (Swarup & Rajeswari, 2007). In tumor diseases, cfDNA levels have been used to assess tumor burden, progression, and response to treatment (Bettegowda et al., 2014; Ehlert et al., 2017; Fleischhacker & Schmidt, 2007; Parkinson et al., 2016). Elevated levels of cfDNA have also been found in patients with severe bacterial infections or viral infections and correlated with disease progression and severity (Ha et al., 2011; Yi et al., 2014). In particular, recent studies (Andargie et al., 2021; Mishra et al., 2022; Scozzi et al., 2021) report on the value of cfDNA as a predictive biomarker for COVID-19 severity. The measurement of cfDNA measurement in patient blood plasma is a highly precise and minimally invasive diagnostic method applied in a wide range of pathological conditions. It can also provide information about the tissue-of-origin through its nucleosome footprint (Snyder et al., 2016; Sun et al., 2019). Here, we present data from a retrospective observational pilot study to further assess the value of cfDNA as a potential biomarker in hospitalized COVID-19 patients during the first wave of infection and compare it to cfDNA levels in a healthy control group.

## Materials and Methods

Portions of this text were previously published as part of a preprint: https://www.medrxiv.org/content/10.1101/2021.04.29.21256291v1.

### Patients

A total of 21 patients hospitalized during the first wave of SARS-CoV-19 infection between March and June 2020 at the University Medical Centre, Mainz, Germany, were evaluated in this retrospective pilot study. This retrospective data collection is limited to the analysis of a highly inhomogeneous patient population due to patient consent for release or availability of their excess material, which also reflects the nature of a pilot study. When patients presented to the hospital with symptoms of COVID-19 disease, a nasopharyngeal swab was taken and tested for the presence of SARS-CoV-2 infection by polymerase chain reaction (PCR) in the in-house virology department. If the test was positive, the patients were included in the pilot study. Exclusion criteria were (1) age less than 18 years, (2) pregnancy, (3) breastfeeding women. Patients’ clinical data and laboratory results were reviewed retrospectively using the electronic hospital information systems (i.s.h.med^®^; SAP, Weinheim, Germany, Nexus Swisslab, Berlin, Germany).

**Figure 1:**
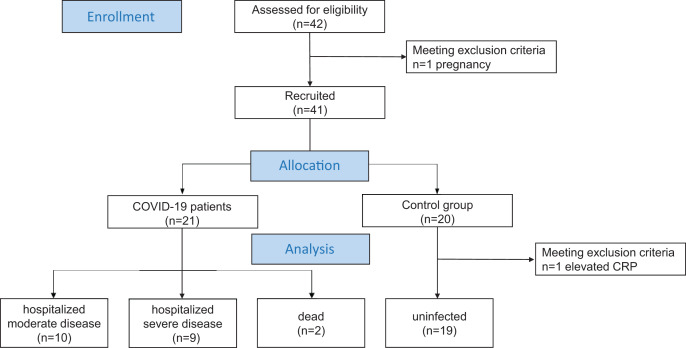
Patient flow diagram. Overview of enrolled patients and controls and grouping according to WHO clinical progression scale.

In addition, a prospective control group (*n* = 19) was included in the study in which SARS-CoV-2 infection was excluded by nasopharyngeal swab. The control group was subject to the exclusion criteria described above. To exclude other acute infections, CRP, leukocytes and platelets were also determined by laboratory tests. The cfDNA levels of the control group were compared with those of the COVID-19 cohort (Fig. 1).

Depending on the severity of disease, patients were classified according to the WHO clinical progression scale (Marshall et al., 2020). It is based on five levels of severity divided into eleven sublevels ranging from 0 (uninfected) to 10 (dead). In this study patients were classified into the five main severity levels according to their worst clinical disease progression, and cfDNA levels were compared between groups. Plasma cfDNA levels were divided into three concentration groups (low ≤ 1,000 ng/mL, moderate 1,000–2,000 ng/mL, and high > 2,000 ng/mL) for correlation with routine clinical laboratory parameters such as CRP, PCT, *etc*., determined in the accredited (DIN-ISO 15.189) Institute for Clinical Chemistry and Laboratory Medicine of the University Medical Centre, Mainz, Germany, as described (Falter et al., 2021).

The pilot study was approved by German law (Landeskrankenhausgesetz §36 and §37) in accordance with the Declaration of Helsinki and by the local ethics committee of the “Landesärztekammer Rheinland-Pfalz” (reference numbers 2020-15116-retrospective). The Ethics Committee waived the requirement for informed consent for the retrospective cohort.

### Blood sample processing and quantification of cfDNA

Lithium-heparin syringes (S-Monovette, Sarstedt) were used to collect blood plasma samples for routine laboratory analysis. Excess blood plasma was centrifuged at 3,746 × g for 10 min at room temperature in a safety level 2 laboratory (S2) within 2–3 h after collection. Plasma aliquots were stored at −80 °C prior to quantification of cfDNA concentrations. Plasma cfDNA concentrations were determined according to Neuberger et al. (2021) using plasma samples and quantitative real-time PCR (qPCR) without prior DNA purification steps. The SYBR Green real-time qPCR amplifies a repetitive 90 bp fragment of human long nuclear elements (LINEs) of the L1PA2 family using the following forward and reverse primers: 5′-TGCCGCAATAAACATACGTG-3′, 5′-GACCCAGCCATCCCATTAC-3′. Plasma was diluted 1:10 in ultra-pure H_2_O (Invitrogen, Carlsbad, CA, USA), added to the reaction mix in a proportion of 1:7.5, and measured in technical replicates in a final volume of 5 µl. The final concentrations of the reaction mix were 0.04 IU Velocity Polymerase (Bioline, London, UK), 1.2 × Hifi Buffer (Bioline, London, UK), 0.15 × SYBR Green (Sigma, St. Louis, MO, USA), 0.3 mM dNTPs (Bioline, London, UK), 140 nM of each primer. Samples were amplified using a Bio-Rad CFX384 system (Bio-Rad, Hercules, CA, USA), using the following protocol: 98 °C for 2 min, followed by 35 cycles of 95 °C for 10 s (denaturation) and 64 °C for 10 s (annealing/extension), followed by a melting curve from 70–95 °C with 0.5 °C increments for 10 s. No template control (NTC) samples, including H_2_O and mouse plasma, and two reference samples for calibration were included in each qPCR run. As described by Neuberger et al. (2021), the linearity, limit of quantification, and limit of detection of the assay, as well as the reliability of the reference sample calibration were determined prior to the measurements (LIT Neuberger et al., 2021). To calculate the amount of DNA, three independent standard curves were prepared to determine the slope, intercept, and dynamic range of the assay. After calibration of the reference samples, the DNA concentration was determined respecting the Cq value of the measured sample, the intercept and slope from the pre-validated assay, the final reaction volume (5 µl), the dilution of the template (1:75), the number of primer targets per human genome (3,416), and the weight per diploid genome (~3.23 pg) using the following formula (ng/mL = pg/µL = 10((Cq-intercept)/slope)/5 µL/(1/75) × 3.23 pg/3,416). The assay has a limit of quantification <1 ng/ml, a sufficient dynamic range and repeatability <12%. To determine storage-dependent changes in cfDNA levels in lithium-heparin syringes, EDTA or lithium-heparin syringes containing plasma from three different subjects were collected and processed immediately, or stored at 4 °C for 5 days. As expected, EDTA samples showed a significant ~60-fold increase in cfDNA concentration during prolonged storage (*p* = 0.004). In lithium-heparin samples, no significant differences were observed between direct processing and extended storage (*p* = 0.5). The samples showed a concentration of 71.8 ± 33.1 and 80.2 ± 32.1 ng/ml (mean ± SD), respectively, demonstrating that lithium-heparin plasma samples are more suitable for cfDNA analysis than EDTA plasma samples.

### Statistical analysis

Data analysis is descriptive. Means and standard deviations are presented for continuous variables, and absolute and relative frequencies and percentages are presented for categorical variables. A two-tailed Student’s T-test was performed to test the hypothesis that cfDNA levels in plasma samples are associated with clinical complications and risk factors in patients. Confidence intervals and *p*-values are reported for the results. Spearman’s rank correlation coefficient was calculated analyzing the laboratory parameters in three different cohorts of cfDNA concentration (low, moderate, high). Spearman’s rho and *p*-values are given.

To test the statistical correlation between the distribution of cfDNA and the classification of patients on the “WHO clinical progression scale”, cfDNA levels were log-transformed and a two-sided Spearman’s rank correlation test was performed. A two-tailed t-test was used to compare the mean values of cfDNA between the control group and each WHO group.

## Results

A total of 21 hospitalized patients with positive evidence of SARS-CoV-2 were evaluated in this pilot study, of whom twelve were male and nine were female. The patients were 68 ± 17 years old and on average overweight (BMI 28.8 ± 6.6 kg/m^2^). The control group consisted of nine female and ten male subjects. The mean age was 43 ± 11 years and differed significantly between the two groups (*p* < 0.001). The control group had a mean BMI of 24.2 ± 4.3, again a significant difference between the groups (*p* = 0.009). There were no significant differences in other patient characteristics between the two groups.

Most of the patients had at least one pre-existing risk factor for a severe course of COVID-19, including cardiac (19%), renal (33%), pulmonary (38%) or immunological (14%) disease, arterial hypertension (57%), diabetes (19%) or adiposity (33%). In terms of pre-existing conditions, the control group had predominantly immunological pre-existing conditions (26.3%), mainly related to the thyroid. Overall, COVID-19 patients had significantly more pre-existing medical conditions than the control group. Patient characteristics are summarized in Table 1.

**Table 1:** Patient and control group characteristics.

Parameters (mean ± SD)	Overall	COVID-19	Control group	*p*-value
Age (years)	56 ± 18.8	68 ± 17	43 ± 11	<0.001
Gender (m/f)	22/18	12/9	10/9	0.78
Height (cm)	174.7 ± 8.7	174.1 ± 8.2	175.3 ± 16.5	0.6
Weight (kg)	81.1 ± 21.3	87.5 ± 22.8	74.1 ± 16.6	0.46
BMI (kg/m^2^)	26.7 ± 6.4	28.8 ± 6.6	23.8 ± 4.1	0.006
Pre-existing conditions (*n* (%))				
Renal	8 (19.5%)	7 (33%)	1 (5.3%)	0.03
Pulmonary	8 (19.5%)	8 (38%)	0 (0%)	0.002
Immunological	8 (19.5%)	3 (14%)	5 (26.3%)	0.36
Arterial hypertension	13 (31.7%)	12 (57%)	1 (5.3%)	<0.001
Cardiac	5 (12.2)	4 (19%)	1 (5.3)	0.2
Diabetes mellitus Type II	4 (9.8%)	4 (19%)	0 (0%)	0.05
Adiposity (BMI ≥ 30 kg/m^2^)	8 (19.5%)	7 (33%)	1 (5.3%)	0.03

**Notes:**

Data are given as mean ± standard deviation and frequency (*n* (%)).

BMI, body mass index; m, male; f, female.

Several of the COVID-19 patients’ clinical laboratory values were elevated during the course of disease (Table 2). Elevated levels of creatinine, urea or LDH and decreased estimated glomerular filtration rate (eGFR) were indicative of organ dysfunction, *i.e*., acute kidney injury (AKI). The inflammatory parameters CRP and procalcitonin (PCT) were markedly present in the majority of COVID-19 patients, reflecting hyperinflammation and/or secondary bacterial infection. Increased D-Dimer concentration was considered as an indicator of COVID-19 associated coagulopathy (Table 2).

**Table 2:** Laboratory parameters reflecting organ dysfunction (creatinine, urea, LDH), inflammation (CRP, PCT) or COVID-19 associated coagulopathy (D-dimer).

Laboratory parameter	Mean ± SD	[Min.–Max.]	Reference range
Maximum D-dimer (mg/l)	7.59 ± 8.09	[0.55–28.69]	<0.50
Maximum creatinine (mg/dl)	2.49 ± 2.23	[0.57–7.24]	0.73–1.18
Maximum urea (mg/dl)	50.71 ± 34.28	[6–106]	9–21
Lowest eGFR (ml/min/m)	50.95 ± 36.29	[7–120]	68–108
Maximum CRP (mg/l)	235.7 ± 158	[46–568]	<5
Maximum PCT (ng/ml)	11.56 ± 43.99	[0.02–208]	<0.5
Maximum LDH (U/l)	669.67 ± 374.5	[235–1,849]	<245

**Note:**

CRP, C-reactive protein; eGFR, estimated glomerular filtration rate; LDH, lactate dehydrogenase; PCT, Procalcitonin; SD, standard deviation.

Patients suffered from various complications, most commonly AKI (62%), followed by pulmonary complications including invasive ventilation and ARDS (43%), anemia and secondary infections (Table 3). COVID-19 was potentially fatal, with 2 of 21 patients dying during hospitalization (Table 4).

**Table 3:** Overview of complications.

Type of complication	Frequency (*n* (%)) (*n* = 21)	Gender distribution (f/m)
Pulmonary complications		
ARDS	9 (43%)	2/7
ECMO therapy	0 (0%)	n.a.
Invasive ventilation ≥7 d	9 (43%)	2/7
Reintubation	2 (11%)	1/1
Tracheostomy	6 (34%)	2/4
Lowest oxygenation-index (mmHg)	98.33 ± 37.65	n.a.
Need for invasive ventilation (days)	20.4 ± 15.51	n.a.
Thromboembolism		
Mesenterial ischemia	1 (5%)	0/1
Catheter associated thrombosis	2 (10%)	0/2
Pulmonary artery embolism	1 (5%)	1/0
Neurological complications		
Critical illness polyneuropathy	1 (5%)	0/1
Delirium	7 (33%)	2/5
Relapse of pre-existing condition	3 (14%)	1/2
Delayed wake up	3 (14%)	0/3
ICU acquired weakness	1 (5%)	1/2
Renal complications		
AKI 1–3 (KDIGO or RRT)	13 (62%)	3/10
Cardiac complications		
CPR	1 (5%)	1/0
Myocardial injury	1 (5%)	0/1
Atrial fibrillation	2 (10%)	0/2
Angina pectoris	1 (5%)	0/1
Other complications		
Secondary infection	12 (57%)	3/9
Anemia	18 (86%)	6/12
Diarrhea	2 (10%)	1/1

**Note:**

AKI, acute kidney injury; ARDS, acute respiratory distress syndrome; CRP, C-reactive protein; ECMO, extra corporal membrane oxygenation; ICU, intensive care unit; KDIGO, kidney disease: improving global outcomes; RRT, renal replacement therapy; SD, standard deviation; f, female; m, male.

**Table 4:** Course of the disease.

Parameter	*n* (%) or mean ± SD	Gender distribution (f/m)
LOS ICU (d)	28.78 ± 19.31	
Readmission on ICU	1 (5%)	1/0
LOS in-hospital (d)	29.94 ± 19.45	
Mortality (*n* (%))	2 (10%)	0/2

**Note:**

d, days; ICU, Intensive care unit; LOS, Length of stay; SD, standard deviation; f, female; m, male.

Table 5 shows the correlation between cfDNA levels and complications, risk factors and outcome parameters. The highest correlation was detected between cfDNA and myositis (*p* = 0.049). Interestingly, thromboembolism was found in patients with lower cfDNA concentrations and higher cfDNA concentrations were associated with absence of thromboembolism. Regarding patient outcome, cfDNA was significantly associated with in-hospital mortality (*p* < 0.001) (Table 5).

**Table 5:** Association between cfDNA concentration and complications, risk factors or outcome.

Complication, risk factor or outcome	cfDNA (ng/ml; mean ± SD)	95% Confidence interval	T value	*p*-value
Complications				
ARDS	2,010 ± 1,068. *n* = 11	[−962.5 to 1,741.6]	0.603	0.554
No ARDS	1,620 ± 1,829. *n* = 10			
Thromboembolism	1,054 ± 689. *n* = 4	[−2,626.3 to 723.3]	−1.189	0.249
No thromboembolism	2,005 ± 1,540. *n* = 17			
AKI	1,679 ± 1,998. *n* = 13	[−1,019.3 to 1,964.4]	0.663	0.515
No AKI	1,914 ± 1,084. *n* = 8			
Vasopressor	1,769 ± 889. *n* = 9	[−1,473.2 to 1,280.2]	−0.147	0.885
No vasopressor	1,866 ± 1,808. *n* = 12			
Delirium	1,741 ± 1,069. *n* = 10	[−1,521.4 to 1,204.2]	−0.244	0.81
No delirium	1,900 ± 1,786. *n* = 11			
Myositis	2,260 ± 1,582. *n* = 14	[4.5–2,609.9]	2.1	0.049
No myositis	953 ± 553. *n* = 7			
SOFA <= 9	1,237 ± 596. *n* = 3	[−1,155.7 to 3,168.5]	1.073	0.314
SOFA > 9	1,853 ± 978. *n* = 7			
Risk factors				
Arterial hypertension	1,744 ± 1,124. *n* = 12	[−1,532 to 1,186.5]	−0.286	0.778
No arterial hypertension	1,932 ± 1,879. *n* = 9			
Diabetes mellitus	1,687 ± 1,183. *n* = 4	[−1,903.1 to 1,565.1]	−0.204	0.869
No diabetes mellitus	1,856 ± 1,542. *n* = 17			
BMI >= 30 BMI < 30	1,724 ± 1,075. *n* = 7 1,874 ± 1,648. *n* = 14	[−1,594.0 to 1,294.6]	−0.217	0.831
Outcome				
Survived	1,470 ± 860. *n* = 20	[2,332.9–5,229.0]	5.47	<0.001
Died	5,245 ± 1,557. *n* = 2			

**Notes:**

Two-sided T-test (unequal variance, Levene). *p*-value, significance level.

AKI, acute kidney injury; ARDS, acute respiratory distress syndrome; BMI, body mass index; cfDNA, cell-free Desoxyribonucleic acid; SOFA, sequential organ failure assessment.

**Figure 2:**
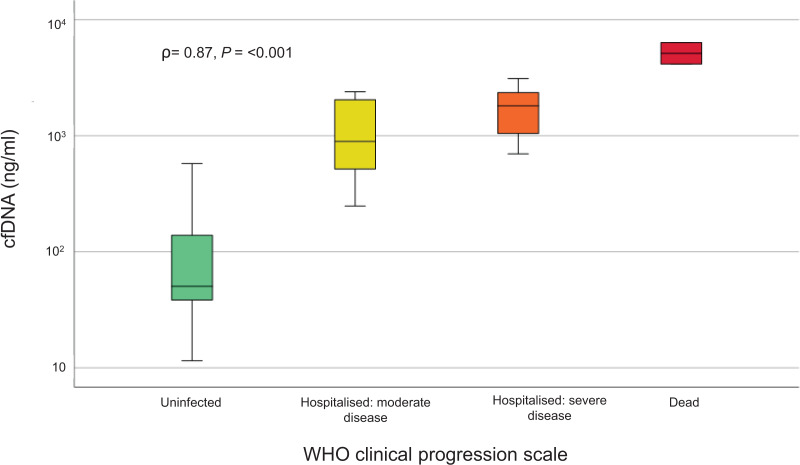
Correlation between cfDNA (ng/ml) and disease severity classification of patients in the treatment and control groups according to the WHO clinical progression scale. Statistical analysis was performed using the two-sided Spearman rank correlation test after logarithmic transformation of the cfDNA levels.

The mean plasma cfDNA concentration in COVID-19 patients was 1,831 ± 1,388 ng/ml (Table 6), with the lowest cfDNA concentration being 248 ng/ml and the highest cfDNA being 6,346 ng/ml. COVID-19 patients and the control group were graded according to the “WHO clinical progression scale”. Patients were classified as “hospitalized with moderate disease”, “hospitalized with severe disease”, and “dead”. Subjects in the control group were classified as “uninfected, no viral RNA detected”. A significant increase in cfDNA levels was observed between the control group and COVID-19 patients (Table 7). cfDNA levels gradually increased with the severity of disease progression from “hospitalized with moderate disease” to “dead” (Fig. 2). The Spearman’s rank correlation coefficient was 0.87 at a significance level of <0.001.

**Table 6:** Groups of cfDNA concentrations in COVID-19 patients.

Cohort	Sample size *n* (%)	Gender distribution (f/m)	Mean ± SD (ng/ml)
All patients	21 (100%)	9/12	1,813.1 ± 1,387.96
cfDNA. high >2,000 ng/ml	8 (36%)	4/4	3,190.1 ± 1,350
cfDNA. moderate 1,000–2,000 ng/ml	7 (32%)	1/6	1,475.6 ± 347.5
cfDNA. low <1,000 ng/ml	7 (32%)	4/3	576.9 ± 204.2

**Note:**

cfDNA, cell-free Desoxyribonucleic acid; SD, standard deviation; f, female; m, male.

**Table 7:** Distribution of cfDNA in the WHO clinical progression scale.

Cohort	Sample size *n* (%)	Gender distribution (f/m)	Mean ± SD (ng/ml)	*p*-value
Control group (uninfected)	19	9/10	108.79 ± 132.1	
COVID-19 patients	21 (100%)	9/12	1,813.1 ± 1,387.96	<0.001
Hospitalized: moderate disease	10 (47.6%)	7/3	1,189.6 ± 824.05	<0.001
Hospitalized: severe disease	9 (42.9%)	2/7	1,769.05 ± 889.5	<0.001
Dead	2 (9.5%)	0/2	5,245.05 ± 1,557.33	<0.001

**Notes:**

cfDNA, cell-free Desoxyribonucleic acid; SD, standard deviation; f, female; m, male.

*p*-values obtained from paired t-test.

Associations between these three groups and complications were predominantly cardiovascular, renal, and pulmonary. Complications were most common in the hospitalized group with severe disease (Table 8). The levels of laboratory parameters correlated with the levels of cfDNA, with CRP and D-dimer showing the highest statistical correlation (*p* = 0.08, Table 9).

**Table 8:** Complications and outcome measured on the WHO clinical progression scale.

Complications	Hospitalized: moderate disease (*n* = 10)	Hospitalized: severe disease (*n* = 9)	Dead (*n* = 2)
Cardiovasculatory	0 (0%)	9 (100%)	0 (0%)
Renal	3 (30%)	9 (100%)	1 (50%)
Pulmonary	1 (10%)	9 (100%)	1 (50%)
Neurological	3 (30%)	6 (67%)	1 (50%)
Thromboembolism	1 (10%)	3 (33%)	0 (0%)
Other complications	5 (50%)	7 (78%)	2 (100%)

**Notes:**

Data are given in *n* (%).

cfDNA, cell-free desoxyribonucleic acid.

**Table 9:** Association between cfDNA concentration and laboratory parameters.

Laboratory parameters (unit, normal range, reported value)	Low cfDNA (*n* = 7, mean)	Moderate cfDNA (*n* = 6, mean)	High cfDNA (*n* = 8, mean)	Spearman’s rho	*p*-vaule
Creatinine (mg/dl, 0.73–1.18, max in 7 days)	0.68	1.77	1.85	0.28	0.21
Bilirubin (mg/dl, 0.2–1.2, max in 7 days)	0.70	1.35	0.75	0.24	0.29
Lactate (mmol/l, 0.5–1.6, max in 2 days)	1.10	1.40	1.90	0.36	0.23
LDH (U/l, <245, max in 2 days)	386.00	367.50	481.50	0.38	0.18
D-dimer (mg/l, <0.5, max in 2 days)	1.12	4.01	4.27	0.67	0.08
Thrombocytes (/nl, 150–360, min in 2 days)	227.00	263.50	230.00	−0.01	0.98
CRP (mg/l, <5, max in 2 days)	54.00	65.50	89.00	0.39	0.08
Leucocytes (/nl, 3.5–10, max in 2 days)	8.30	8.67	8.54	0.10	0.66

**Notes:**

cfDNA, cell-free desoxyribonucleic acid; CRP, C-reactive protein; LDH, lactate dehydrogenase.

Spearman’s rank correlation coefficient was calculated analyzing the laboratory parameters in three different cohorts of cfDNA concentration (low, moderate, high). The laboratory parameters evaluated here are maximum (max) or minimum (min) values during the next 2 or 7 days after cfDNA concentration was measured.

## Discussion

This retrospective observational pilot study compared plasma cfDNA levels between COVID-19 patients and an uninfected control group and explored correlations between cfDNA levels and clinical complications and outcomes as well as clinical blood laboratory measures in patients with COVID-19 during the first wave of infection. In our patient cohort, cfDNA levels were associated with patient outcome as measured by the WHO clinical progression scale, mortality, complications such as myositis, elevated D-dimer and CRP levels, thus reflecting outcome, organ complications and inflammation. Limitations of the present pilot study include the small cohort size with a broad spectrum of COVID-19 severity. This is mainly due to the unique human resource and structural conditions at the beginning of the COVID-19 pandemic. In such exceptional situations, the evaluation of small patient cohorts is extremely important in order to gain relevant clinical insights for future studies and therapy. We also analyzed a single plasma sample from each patient at individually different stages of their hospitalization. Therefore, it is not possible to generalize the results of this pilot study to all patients with COVID-19 and we cannot discuss on dynamic alterations of cfDNA data over time. Nevertheless, this pilot study has shown that even in a small sample, cfDNA levels correlate with disease severity as measured by the “WHO clinical progression scale”, clinical laboratory parameters and complications, and are significantly different from uninfected controls.

Pre-existing conditions other than hypertension, obesity, and diabetes mellitus, which are considered typical risk factors for severe COVID-19 disease, were not assessed in this study. Although these pre-existing conditions were not correlated with cfDNA levels, we cannot exclude the possibility that other pre-existing conditions may have an overall effect on cfDNA levels that was not captured in this pilot study.

cfDNA has been shown to be a valid marker for acute conditions such as stroke (O’Connell et al., 2017). Other pre-existing conditions, such as cardiovascular disease, can also affect baseline cfDNA levels (de Miranda et al., 2021). For example, a recent myocardial infarction resulted in 10-fold higher cfDNA levels compared to a healthy control group (Einbinder et al., 2020). Data on the impact of chronic pre-existing conditions are scarce. However, several factors may contribute to its increase, such as hypertension, smoking, diabetes mellitus, physical activity, ageing and other unknown factors (de Miranda et al., 2021).

Notably, our assay amplifies repetitive LINE-1 elements, which comprise approximately 17% of the human genome. LINE-1 are retrotransposable elements that are epigenetically silenced in somatic cells and exhibit rare events of retrotransposition (Brouha et al., 2003; Xiao-Jie et al., 2016). LINE-1 dysregulation has been described in diseases such as cancer, but also metabolic, neurological and autoimmune disorders (Zhang, Zhang & Yu, 2020), which could affect the number of targeted LINE-1 elements.

Currently, there is no universal protocol for measuring cfDNA levels and presenting the data (Andargie et al., 2021; de Miranda et al., 2021; Zuo et al., 2020). This limits the comparability of studies and their results.

In addition, our results confirm previous studies showing that cfDNA is a liquid biopsy marker potentially suitable for predicting disease severity in patients with COVID-19 (Andargie et al., 2021; Mishra et al., 2022; Zuo et al., 2020). Another limitation of our retrospective pilot study is, that we examined cfDNA levels from patients hospitalized during the first wave of SARS-CoV-2 infection. Subsequent emergence of variants of concern (VOC) or vaccination may have affected plasma cfDNA levels in patients with SARS-CoV-2 during the more recent waves of infection. However, apart from the aforementioned pre-existing conditions, the absence of vaccines and VOCs at the time of sample collection provides a fairly unbiased picture of cfDNA levels in COVID-19 patients, but at the same time precludes an unbiased comparison with cfDNA levels from patients who were infected with later emerging VOCs and who may have been vaccinated. Larger prospective studies are needed to analyze the impact of subsequent viral variants on cfDNA levels.

Despite these limitations, our results are consistent with recently reported data on the predictive value of circulating (mitochondrial) cfDNA for COVID-19 outcome (Andargie et al., 2021; Hammad et al., 2021; Scozzi et al., 2021). Significant correlations have also been reported between total cfDNA and LDH (Cheng et al., 2021), a biomarker of cell damage, similar to circulating cfDNA. LDH is commonly elevated in severe COVID-19 cases and is increased in non-survivors (Zhou et al., 2020). As the relative range of cfDNA concentrations found in COVID-19 patients in our pilot study was wider than the range of LDH, cfDNA may be used to more accurately differentiate between different levels of COVID-19 severity and potentially provide a prognostic assessment. Testing this hypothesis is the subject of ongoing studies.

We found the most significant correlations between cfDNA and CRP. Compared to CRP, cfDNA is a potentially more sensitive marker for monitoring and predicting disease progression. CRP is a non-specific acute-phase protein, produced by the liver in response to inflammation and tends to peak within 48 h of symptom onset. The half-life of CRP ranges from 12 to 24 h, depending on the underlying disease and factors such as age. On the other hand, cfDNA is released immediately after cell damage and may be useful in monitoring disease severity, progression, treatment response and predicting outcome in infectious diseases. The half-life of cfDNA in healthy individuals ranges from 15 min to 2 h, and can be increased in various health conditions, allowing for “real-time” analysis (Kustanovich et al., 2019). Furthermore, cfDNA levels show a wide range between 0 and 100 ng/ml in healthy individuals and between 0–5 and >1,000 ng/ml in cancer patients (Thierry et al., 2016). In contrast, CRP shows a narrower range. CRP levels >500 mg/l are very rare. Therefore, cfDNA is likely to allow finer graded differentiation for diagnosis and prognosis.

Although both CRP and cfDNA are non-specific markers, cfDNA can provide information on the origin of cell damage through methylation analysis to specify organ damage.

With our research showing that cfDNA correlates with disease severity in SARS-CoV-2 infection, further studies can be initiated to substantiate this advanced hypothesis.

**Figure 3:**
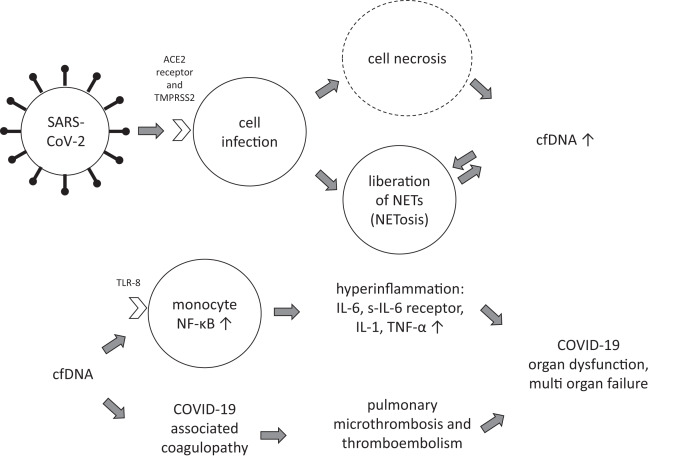
Hypothetical role of cfDNA in COVID-19. ACE2 receptor, angiotensin-converting enzyme 2 receptor; cfDNA, cell-free desoxyribonucleic acid; IL, interleukin; NET, neutrophil extracellular traps; NF-κB, nuclear factor kappa B; TLR, toll-like receptor; TMPRSS2, transmembrane protease serine subtype 2; TNF-α, tumor necrosis factor alpha.

There are still hurdles to overcome before cfDNA can be used as a full-fledged biomarker for infectious diseases. Despite years of implementation in many medical fields, the determination of cfDNA is still very time-consuming compared to conventional inflammation parameters. The measurements are still not fully automated and require a high level of experience and expertise from laboratory professionals. In infectious disease monitoring, a combination of multiple biomarkers provides the best assessment of disease progression and response to treatment. CfDNA can add significant value to this portfolio. It should be noted that cfDNA is not only a biomarker for upstream pathophysiological mechanisms, but has also been proposed to trigger specific downstream effects. For example, cfDNA has been shown to be a regulator of the immune system (Korabecna et al., 2020), with different immunoregulatory properties in healthy and diseased individuals (Brynychova et al., 2019). It has also become clear that cfDNA is one of the factors contributing to the formation of neutrophil extracellular trap (NET). NET plays a key role in immunothrombosis and has been shown to be consistently increased in COVID-19 and associated with disease severity (Leppkes et al., 2020; Ng et al., 2020). Currently, NET is being discussed as a potentially useful biomarker to discriminate between severe and non-severe COVID-19, but not to predict thrombotic risk (Gorog et al., 2022). Indeed, complications such as acute arterial thromboembolism have been reported in COVID-19 (Indes et al., 2021), and inflammatory cells are prominent in arterial thromboembolic material from COVID-19 patients (Yesilkaya et al., 2021). However, recent data have shown no evidence of classic thrombotic microangiopathy in COVID-19 (Falter et al., 2021), consistent with our observation that higher cfDNA concentrations were not associated with thromboembolism. One hypothesis is that laboratory features of thrombotic microangiopathy are absent in COVID-19 due to its limitation to the pulmonary microcirculation (Falter et al., 2021), thereby distinguishing the pulmonary pathology of COVID-19 from that of an equally severe influenza virus infection (Ackermann et al., 2020). It has been hypothesized that neutrophils may amplify pathological damage and exacerbate a hyperinflammatory state (Borges et al., 2020). SARS-CoV-2 was also found to induce the release of neutrophil extracellular traps (NETs) by neutrophils (Veras et al., 2020). It remains to be clarified whether specific pathways downstream of cfDNA are critical for hyperinflammation and COVID-19-associated coagulopathy following SARS-CoV-2 infection. However, current evidence is consistent with the hypothesis that cfDNA triggers both hyperinflammation and coagulation (Fig. 3). Given that pre-existing conditions also affect cfDNA levels, this may contribute to the exacerbation of the acute infection in pre-diseased patients.

D-dimer is another biomarker for of coagulation and fibrinolysis (Krychtiuk et al., 2021) that is elevated in patients with COVID-19 (Berger et al., 2020). The consensus statement of the COVID-19 International Colloquium on Thrombosis confirms that D-dimer is associated with disease severity and adverse outcomes (Gorog et al., 2022). Other studies have shown a positive association between elevated CRP levels and disease severity and mortality in COVID-19 (Leisman et al., 2020; Zhou et al., 2020). Our data are in line with these findings. D-dimer and CRP levels were the most highly correlated with cfDNA levels in this pilot study.

## Conclusions

CfDNA levels in blood plasma samples from COVID-19 patients were significantly higher than those in an uninfected control group and correlated with the occurrence of clinical complications and disease severity as measured by the “WHO clinical progression scale”. Clinical laboratory measurements of D-dimer and CRP showed the highest correlations with cfDNA levels. The strong elevation of cfDNA in patients with COVID-19 in the first wave of disease confirms that cfDNA may be an important prognostic factor. This association has also been demonstrated in later waves of infection. However, inconsistent quantification methods make it difficult to compare measurements across studies. Whether cfDNA is a suitable method for monitoring therapy during the course of disease needs to be investigated in further studies. Therefore, prospective studies with quantification of cfDNA levels at defined time points during the course of disease are required.

## Supplemental Information

10.7717/peerj.16072/supp-1 Supplemental Information 1 CONSORT checklist.

10.7717/peerj.16072/supp-2 Supplemental Information 2 Raw data. The patients’ laboratory parameters, comorbidities, duration of hospital- and ICU-stay and cfDNA measurements.

10.7717/peerj.16072/supp-3 Supplemental Information 3 Study protocol.

## Additional Information and Declarations

### Data Availability

The following information was supplied regarding data availability: The raw data is available in the Supplemental File.

### Clinical Trial Registration

The following information was supplied regarding Clinical Trial registration: DRKS00029389.
