# Supplementary material for: Evidence for the utility of cfDNA plasma concentrations to predict disease severity in COVID-19: a retrospective pilot study
Source: PeerJ. 2023 Sep 18;11:e16072. doi: 10.7717/peerj.16072 (PMC10512938; doi:10.7717/peerj.16072)
Supplement: Supplemental Information 3 [file peerj-11-16072-s003.docx]

**Evidence for the utility of cfDNA plasma concentrations to predict disease severity in COVID-19: A pilot study**

| Title | Evidence for the utility of cfDNA plasma concentrations to predict disease severity in COVID-19: A pilot study |
| --- | --- |
| Study management | PD Dr. med. Marc Bodenstein |
| Indication | Correlation of measured cfDNA in blood with clinical parameters in patients with COVID-19 disease. |
| Objectives and endpoints | 1. Recording of standard laboratory parameters collected during treatment:    1. LDH    2. Thrombocytes    3. D-Dimer    4. Leukocytes    5. Interleukin 6    6. CRP    7. PCT    8. Creatinine    9. Bilirubin    10. Lactate 2. Recording of personal data:    1. Age    2. Gender    3. Weight    4. Size    5. Pre-existing conditions       1. Cardiac       2. Renal       3. Pulmonary       4. Immunological       5. COVID-19-Trias: arterial hypertension, diabetes mellitus type II, obesity 3. Recording of complications occurring during course of disease:    1. Thromboembolic complications    2. Pulmonary complications (ARDS) defined as:       1. Requirement of mechanical ventilation       2. Bipulmonary infiltrates       3. Minimum Horowitz index       4. ECMO therapy    3. Renal complications with / without RRT    4. Cardiovascular complications with / without catecholamine therapy and / or resuscitation    5. Neurological complications (delirium)    6. Long-term mechanical ventilation with need for tracheostomy or re-intubation    7. Other complications (e.g., rhabdomyolysis, myositis, etc.) 4. Recording of the minimum and maximum SOFA score during intensive care stay 5. Recording of the length of ICU-stay 6. Recording of readmission to ICU 7. Recording of hospital-stay 8. Recording of in-hospital mortality |
| Study design | Monocentric, retrospective data acquisition |
| Study population | In the context of the COVID-19 pandemic, cfDNA was measured experimentally in excess biomaterial of blood samples of 22 patients at freely chosen time points in March, April and May 2020. Clinical outcome parameters (as described in objectives and endpoints) will now be correlated with these measurements. |
| Duration of study | Data collection will be completed by the end of July 2020 |
| Number of participants | The number of patients included in the study is 22. |
| Data resources | • Routinely collected patient records during hospital stay  • Routine laboratory parameters collected during hospital stay  • Routine radiological findings obtained during hospital stay  • cfDNA level obtained from patients’ blood samples |
| Data collection | • Collection of pseudonymized data from data sources  • Statistical consulting after data collection |
